# Energy and Electronic Properties of Nanostructures Based on the CL-20 Framework with the Replacement of the Carbon Atoms by Silicon and Germanium: A Density Functional Theory Study

**DOI:** 10.3390/ma15196577

**Published:** 2022-09-22

**Authors:** Margarita A. Gimaldinova, Mikhail M. Maslov, Konstantin P. Katin

**Affiliations:** 1 Nanoengineering in Electronics, Spintronics and Photonics Institute, National Research Nuclear University “MEPhI”, Kashirskoe Shosse 31, 115409 Moscow, Russia; 2 Laboratory of Computational Design of Nanostructures, Nanodevices, and Nanotechnologies, Research Institute for the Development of Scientific and Educational Potential of Youth, Aviatorov Str. 14/55, 119620 Moscow, Russia

**Keywords:** high-energy molecules, CL-20, density functional theory, chemical reactivity, strain energy, covalent dimers

## Abstract

We consider Si*_n_*CL-20 and Ge*_n_*CL-20 systems with carbon atoms replaced by silicon/germanium atoms and their dimers. The physicochemical properties of the silicon/germanium analogs of the high-energy molecule CL-20 and its dimers were determined and studied using density functional theory with the B3LYP/6-311G(d,p) level of theory. It was found that the structure and geometry of Si*_n_*CL-20/Ge*_n_*CL-20 molecules change dramatically with the appearance of Si-/Ge-atoms. The main difference between silicon- or germanium-substituted Si*_n_*CL-20/Ge*_n_*CL-20 molecules and the pure CL-20 molecule is that the NO_2_ functional groups make a significant rotation relative to the starting position in the classical molecule, and the effective diameter of the frame of the systems increases with the addition of Si-/Ge-atoms. Thus, the effective framework diameter of a pure CL-20 molecule is 3.208 Å, while the effective diameter of a fully silicon-substituted Si_6_CL-20 molecule is 4.125 Å, and this parameter for a fully germanium-substituted Ge_6_CL-20 molecule is 4.357 Å. The addition of silicon/germanium atoms to the system leads to a decrease in the binding energy. In detail, the binding energies for CL-20/Si_6_CL-20/Ge_6_CL-20 molecules are 4.026, 3.699, 3.426 eV/atom, respectively. However, it has been established that the framework maintains stability, with an increase in the number of substituting silicon or germanium atoms. In addition, we designed homodesmotic reactions for the CL-20 molecule and its substituted derivatives Si_6_CL-20/Ge_6_CL-20, and then determined the strain energy to find out in which case more energy would be released when the framework breaks. Further, we also studied the electronic properties of systems based on CL-20 molecules. It was found that the addition of germanium or silicon atoms instead of carbon leads to a decrease in the size of the HOMO–LUMO gap. Thus, the HOMO–LUMO gaps of the CL-20/Si_6_CL-20/Ge_6_CL-20 molecules are 5.693, 5.339, and 5.427 eV, respectively. A similar dependence is also observed for CL-20 dimers. So, in this work, we have described in detail the dependence of the physicochemical parameters of CL-20 molecules and their dimers on the types of atoms upon substitution.

## 1. Introduction

**Figure 1:**
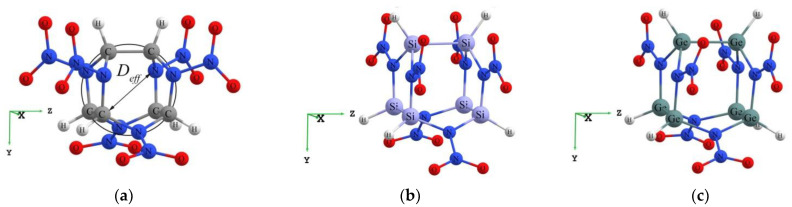
(**a**)—The isolated molecule CL-20; (**b**)—the isolated molecule Si_6_CL-20; (**c**)—the isolated molecule Ge_6_CL-20.

The isolated molecule CL-20, also known as hexanitrohexaazaisowurtzitane (C_6_H_6_N_12_O_12_), was synthesized in California in 1987 [1]. It is representative of the class of high-energy-density materials (HEDMs) [2], and its unique properties are due to its unusual size and shape. Isolated CL-20 is formed by a strained carbon–nitrogen framework consisting of two five-membered and one six-membered ring connected by C-C bonds and six NO_2_ functional groups attached to this framework. Figure 1a illustrates the molecular structure of the C_6_H_6_N_12_O_12_. Hexanitrohexaazaisowurtzitane (CL-20) is the most promising HEDM at present and exhibits better comprehensive properties than most high-energy materials [3].

Previously, nanostructures based on hexanitrohexaazaisowurtzitane were created by various methods to improve the properties of these compounds, such as composition density, thermal stability, and others. Despite the fact that CL-20 is a metastable compound, it can form molecular crystals, in which separate CL-20 molecules retain their individuality [4,5,6]. Moreover, currently, the cocrystallization method is widely applied to various high-energy materials including CL-20. A large number of molecular cocrystals based on CL-20 were obtained, and their properties were studied, for example, TNT/CL-20 [7,8,9,10], HMX/CL-20 [7,8], CL-20/DNT [11], CL-20/RDX [12], etc.

Then, for the first time, a molecule-based covalent CL-20 crystal was predicted using computer simulation. One-dimensional CL-20 chains have been constructed using CH_2_ molecular bridges for the covalent bonding between the isolated CL-20 fragments. It was found that such systems become more thermodynamically stable as their efficient length increases [13]. It is confirmed that the use of CH_2_ bridges is the universal technique to connect both CL-20 fragments in the chain and the chains together to make a network. In the previous study [14], we analyzed the properties of the quasi-one-dimensional and quasi-two-dimensional systems consisting of CL-20 molecules. Covalent crystals have several advantages over molecular ones since they have a higher density along with higher kinetic stability. Thus, the formation of bulk covalent CL-20 solids may be energetically favourable.

Furthermore, it is known that carbon-based compounds can successfully exist even if carbon atoms are replaced by their closest analogues in the periodic table—silicon and germanium. Silicon and germanium are similar to carbon in some chemical properties. The successful replacement of carbon atoms with silicon and germanium atoms is found in various applications. Thus, organic cells with carbon-silicon chemical bonds were obtained using a live biocatalyst [15]. In research on new drugs, carbon is replaced by silicon [16]. Chemistry also uses organic polymers based on silicon [17]. They are used in agriculture and the semiconductor industry. Another example of a successful replacement of carbon atoms by silicon atoms is silicon polyprismanes. Silicon polyprismanes are nanotubes of a particular type, built from silicon rings. It was found that such nanostructures exhibit unique metallic properties [18]. Also, in [19] the geometrical structure and nuclear magnetic resonance (NMR) parameters of the pristine, as well as carbon-, silicon-, and germanium-doped (10,0) boron nitride (BN) nanotubes, have been studied using a DFT/B3LYP method for the first time. It was found that the NMR parameters are significantly changed for those B and N nuclei that bond directly to C, Si, or a Ge dopant. Thus, compounds based on silicon and germanium may have unusual characteristics, and the substitution of carbon atoms leads to improving the properties of such systems. This approach is also applied to CL-20 molecules [20,21].

In this paper, density functional theory simulations were performed to predict the stability, energetic performance, and the physical and chemical properties of the CL-20 systems, wherein carbon atoms are replaced by silicon or germanium atoms. Density functional theory (DFT) is one of the most accurate methods to identify molecules’ specifications and characteristics and is widely used in modern calculations to determine the properties of carbon systems. For example, in [22], the electronic, magnetic, and optical properties of graphene in the presence of Fe impurities and vacancies are demonstrated using DFT calculation within GGA. It has been found that the conductive property of graphene remains in the presence of Fe atoms and vacancies in the graphene structure. Furthermore, in [23], the authors study the influence of intermolecular interactions on the properties of carbon nanotubes using DFT. The DFT calculations are consistent with experimental data and predict the stability of the systems considered in Ref. [23]. In [24], the molecular adsorption of hydrogen sulfide was investigated theoretically by using density functional theory for gallium (Ga)-, germanium (Ge)-, and boron (B)-doped (4,0) single-walled carbon nanotubes.

Thus, the abbreviated names of systems in the present work are Si*_n_*CL-20/Ge*_n_*CL-20, where *n* is the number of substituted atoms, *n* = 1, …, 6. The fully silicon and germanium substituted molecules Si_6_CL-20/Ge_6_CL-20 are depicted in Figure 1b,c. We explained how the physicochemical properties of systems, such as the binding energy, HOMO–LUMO gap, chemical potential, and others depend on the number of silicon or germanium atoms added to the system instead of carbon. Furthermore, we estimated the cage strain energy of the classical CL-20 molecule using the design of homodesmotic reactions, as well as the effect of the substitution of carbon atoms by silicon and germanium atoms in the compound on this value.

## 2. Computational Details

The structural optimization and characterization of the Si*_n_*CL-20/Ge*_n_*CL-20 systems were carried out with the framework of density functional theory (DFT). Density functional theory is a basic method for the analysis of the properties of low-dimensional nanostructures with highly strained frameworks [25,26] and has been successful in terms of computing speed and accuracy [27].

All DFT calculations for the silicon/germanium analogues of the high-energy molecule CL-20 and its dimers were performed using the TeraChem software [28,29,30,31]. The traditional Becke’s three-parameter hybrid method and the Lee–Yang–Parr exchange–correlation energy functional (B3LYP) [32,33] with the Pople basis set 6-311G(d,p) [34] were used for the calculations.

During geometry optimization, the global charge of all of the systems considered was neutral. The maximum force and root mean square forces were 4.5 × 10^−4^ and 3 × 10^−4^ (hartrees per bohr and hartrees per radian), whereas the maximum displacement and root mean square displacement were 1.8 × 10^−3^ and 1.2 × 10^−3^. Mulliken charges were calculated at the same B3LYP/6-311G(d,p) level of theory. According to a benchmarking study [35], the typical error of energies of high-strained molecules calculated with the used DFT method is about 0.1 eV, and errors in bond lengths are smaller than 0.01 Å [36].

## 3. Results and Discussion

### 3.1. Geometry Optimization

**Figure 2:**
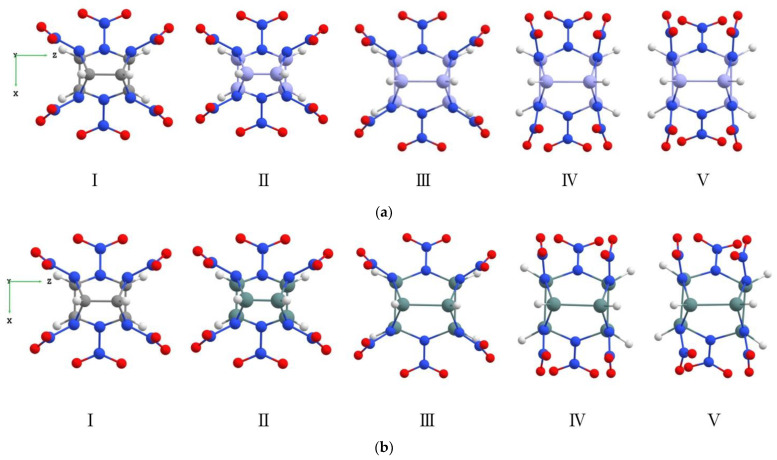
(**a**) From pure CL-20 to substituted Si_6_CL-20; (**b**) From pure CL-20 to substituted Ge_6_CL-20. I—The isolated molecule CL-20 (top view); II—the isolated non-optimized molecule Si_6_CL-20/Ge_6_CL-20 (Si-/Ge-atoms replaced all C-atoms); III, IV—optimization steps of Si_6_CL-20/Ge_6_CL-20 (increase in diameter and rotation of functional groups NO_2_); V—the isolated optimized molecule Si_6_CL-20/Ge_6_CL-20. The color codes of the atoms are the same as in Figure 1.

First of all, we considered the pure unsubstituted CL-20 molecule, and then we began to replace carbon atoms with silicon or germanium atoms. We constructed several types of Si*_n_*CL-20/Ge*_n_*CL-20 molecules, where *n* = 1, …, 6 is the number of silicon/germanium atoms in the system that replaced the carbon atoms (*n* = 0 for traditional CL-20). It should be noted that the structure and geometry of the Si*_n_*CL-20/Ge*_n_*CL-20 molecule change strongly with the appearance of Si- or Ge-atoms in it. It can be seen in Figure 2, wherein the transformations of a pure molecule CL-20 into a fully silicon-substituted molecule Si_6_CL-20 (a) or a fully germanium-substituted molecule Ge_6_CL-20 are shown (b).

The main difference between a pure molecule CL-20 and silicon or germanium molecules Si*_n_*CL-20/Ge*_n_*CL-20 is that the functional groups NO_2_ make a significant rotation (Figure 2a,b) relative to the initial position in the classic molecule (Figure 2aI), and the effective diameter of the framework of the systems increases with the addition of Si-/Ge-atoms.

Table 1 shows the effective diameters for the most thermodynamically stable systems obtained at the B3LYP/6-311G(d,p) level of theory (see the next section for more information on the thermodynamic stability of systems). The effective diameter of the nanostructures was determined by us with the following formula

(1) $$D_{eff}=\frac{\sum_{i=1}^{48}r_{i}}{48},\;$$

where $r_{i}$ is the distances between atoms in the carbon–germanium–nitrogen frame (we took into consideration four maximum distances for each of the 12 atoms included in the carbon–germanium–nitrogen framework of the molecule).

From Table 1, one can see that, when silicon or germanium atoms are added to the system, the effective diameter of the framework increases. It should be noted that an increase in the effective diameter is more significant for germanium-substituted molecules.

Besides, the average bond lengths between carbon, nitrogen, and silicon/germanium atoms, which are part of the framework of the most thermodynamically stable compounds Si*_n_*CL-20/Ge*_n_*CL-20 (*n* = 1,…,6), were measured. These data are presented in Table 2 and Table 3.

### 3.2. Energy Properties

For understanding the energy efficiency and thermodynamic stability of the nanostructures, the binding energies *E_b_* of classic CL-20 and Si*_n_*CL-20/Ge*_n_*CL-20 systems for all possible configurations with *n* = 1, …, 6 silicon or germanium atoms (that replaced the carbon atoms in the traditional CL-20 molecule) were obtained and analyzed. The binding energy *E_b_* of the nanostructure per atom is determined by the equation

(2) $$E_{b}\left[\frac{\mathrm{eV}}{\mathrm{atom}}\right]=\frac{1}{N_{at}}\left\{iE(H)+kE(O)+lE(N)+mE(C)+nE\left(\mathrm{Si}/\mathrm{Ge}\right)-E_{tot}{(\mathrm{Si}}_{n}\mathrm{CL}-20/{\mathrm{Ge}}_{n}\mathrm{CL}-20)\right\}$$

where *N*_at_ = *i* + *k* + *l* + *m* + *n* = 36 is the total number of atoms in the system (*i* = 6; *k* = 12; *l* = 12; *m* + *n* = 6); *E_tot_*(Si_n_CL-20/Ge_n_CL-20) is the total Si*_n_*CL-20/Ge*_n_*CL-20 nanostructure energy; and *E*(H), *E*(O), *E*(N), *E*(O), *E*(Si/Ge) are the energies of the isolated hydrogen, oxygen, nitrogen, carbon, and silicon/germanium atoms, respectively.

**Figure 3:**
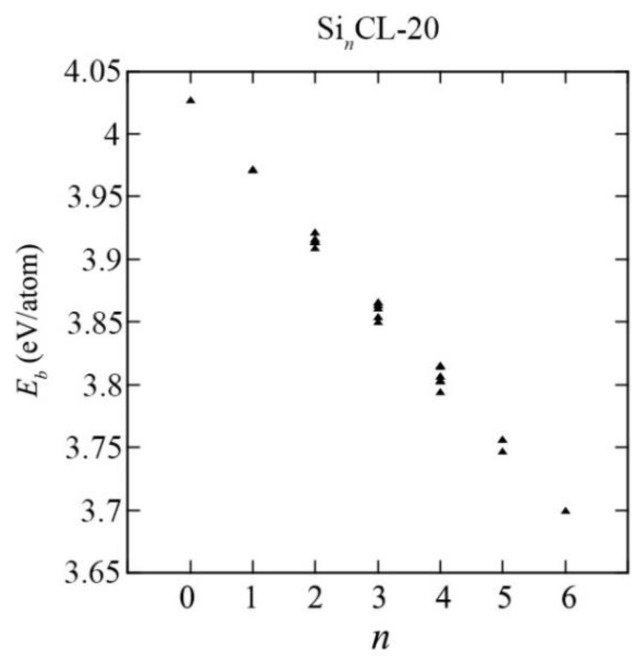
The binding energy versus the number of Si-atoms *n* in the Si_n_CL-20 molecule for all possible configurations obtained at the DFT/B3LYP/6-311(d,p) level of theory.

The system with higher binding energy (lower potential energy) is more thermodynamically stable and vice versa. The binding energies *E_b_* obtained for all possible configurations Si*_n_*CL-20/Ge*_n_*CL-20, where *n* is the number of germanium atoms in the system (*n* = 1,…,6), are presented in Figure 3. The values of the binding energies for all such systems considered can be found in the Supplementary Materials (Tables S1 and S2). The point *n* = 0 corresponds to a pure unsubstituted CL-20 molecule. It can be seen that the addition of silicon/germanium atoms to the system leads to a decrease in the binding energy. Even for *n* = 1, the binding energy is quite noticeably different from the binding energy of a traditional CL-20 molecule. However, it has been established that the framework maintains stability with an increase in the number of substituting silicon or germanium atoms.

**Figure 4:**
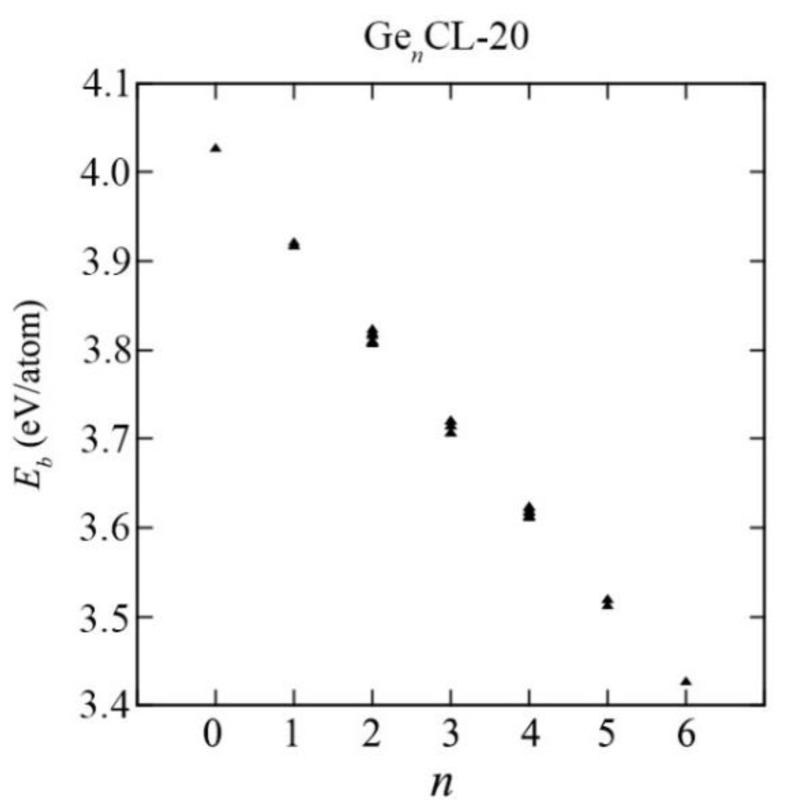
The binding energy versus the number of Ge-atoms *n* in the Ge_n_CL-20 molecule for all possible configurations obtained at the DFT/B3LYP/6-311(d,p) level of theory.

As evident from Figure 3 and Figure 4, as the number of Si-/Ge-atoms *n* in the Si*_n_*CL-20/Ge*_n_*CL-20 molecule increases, the value of the binding energy becomes smaller. Moreover, for germanium-substituted Ge*_n_*CL-20 molecules, the decrease in the binding energy is sharper than that of silicon-substituted Si*_n_*CL-20, and for the same number of substituted atoms *n*, the binding energy of the germanium molecule is less than the corresponding value for the silicon molecule.

**Figure 5:**
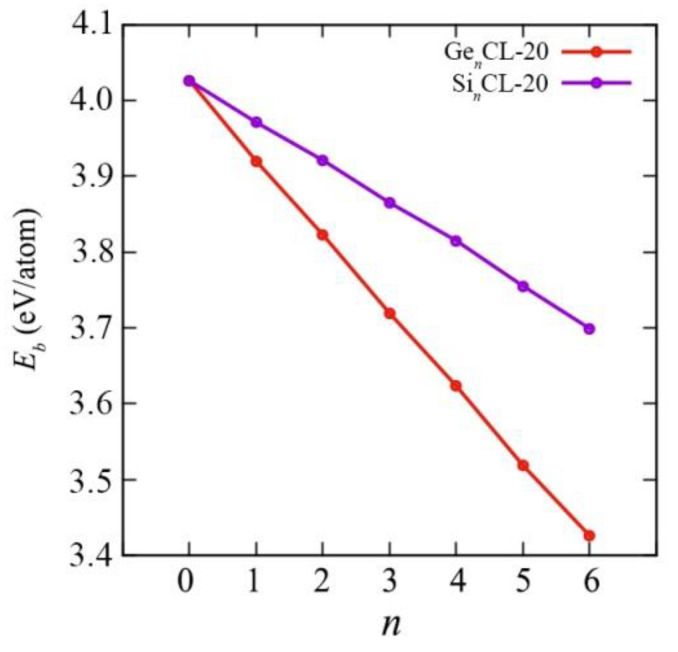
The binding energy versus the number of Si-/Ge-atoms *n* in the SinCL-20/GenCL-20 molecule for the most thermodynamically stable configurations obtained at the DFT/B3LYP/6-311(d,p) level of theory.

For the same number of substituted atoms, the binding energy *E_b_* of the systems is slightly different for various combinations of such atoms. For further consideration in this study, a combination with the maximum binding energy *E_b_* was chosen for each number of substituted atoms *n*, since such systems are the most thermodynamically stable (see Figure 5).

For the maximum binding energies of germanium and silicon CL-20, using the least-squares method, linear regression equations were obtained depending on the number of substituted atoms *n*= 0,..., 6:

*E*_b_(Si_n_CL-20) = −0.054*n* + 4.0273, (3)

*E*_b_(Ge_n_CL-20) = −0.1001*n* + 4.0226. (4)

Thus, we determined all further characteristics specifically for these most thermodynamically stable configurations for each value of Si-/Ge-atoms *n*, because these atomic arrangements are most beneficial. In addition, the data obtained for all possible types of arrangement of the silicon/germanium and carbon atoms in the skeleton of the molecules Si*_n_*CL-20/Ge*_n_*CL-20 for each number of substituted atoms *n* separately are presented in the Supplementary Materials (Tables S1 and S2).

### 3.3. Electronic Characteristics

**Figure 6:**
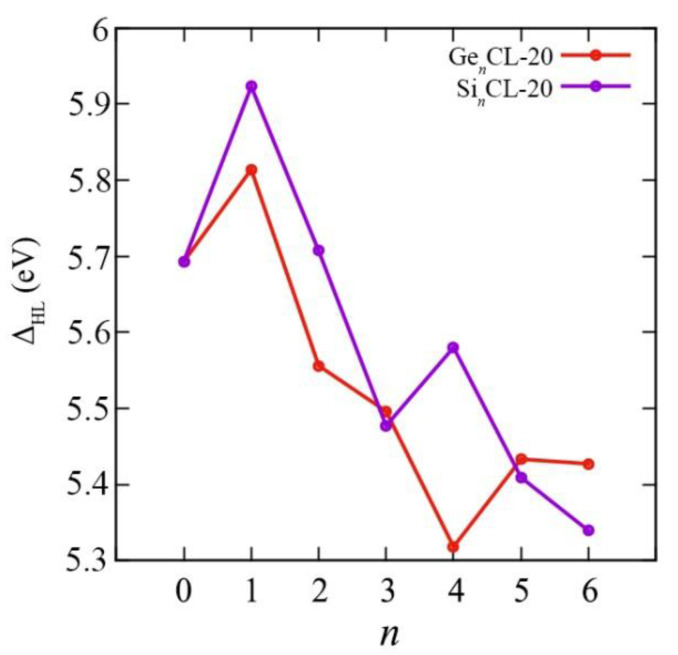
The HOMO–LUMO gap versus the number of Si-/Ge-atoms *n* in the Si*_n_*CL-20/Ge*_n_*CL-20 molecule for the most thermodynamically stable configurations obtained at the DFT/B3LYP/6-311(d,p) level of theory.

To assess the potential use of the Ge*_n_*CL-20 nanostructures in various electronic applications, the HOMO–LUMO gaps of such systems were determined. The HOMO–LUMO gap Δ_HL_ is defined as the energy gap between the highest occupied molecular orbital and the lowest unoccupied molecular orbital. The obtained results for HOMO–LUMO gaps of the most thermodynamically stable systems are presented in Figure 6. The HOMO and LUMO energies themselves of all Si*_n_*CL-20/Ge*_n_*CL-20 nanostructures considered can be found in the Supplementary Materials (Tables S1 and S2).

As is evident from Figure 6, the HOMO–LUMO gap for the Si_1_CL-20 and Ge_1_CL-20 molecules (5.923 eV and 5.814 eV, respectively) is slightly larger than the corresponding value for the unsubstituted CL-20 molecule (5.693 eV). However, when more carbon atoms are replaced by silicon or germanium atoms, the gap size decreases. However, a uniform decrease in this value is violated at *n* = 4. For example, for germanium systems, the Ge_4_CL-20 molecule is characterized by the smallest HOMO–LUMO gap equal to 5.318 eV, while, for silicon systems, at *n*= 4, there is a sharp jump up to 5.580 eV.

Mulliken charge analysis reveals that the largest charge is concentrated on silicon/germanium and nitrogen atoms of the Si*_n_*CL-20/Ge*_n_*CL-20 nanostructure. The greatest positive charge accumulates on silicon/germanium atoms and the greatest negative charge accumulates on nitrogen atoms belonging to the carbon-silicon/germanium-nitrogen framework. Therefore, the caged silicon/germanium atoms are favorable sites for a nucleophilic reaction, whereas the caged nitrogen atoms are a good target for an electrophilic reaction.

### 3.4. Chemical Reactivity Indices

Density functional theory was used in this study for the analysis of the chemical reactivity indices of the compounds and for obtaining a set of physical quantities, such as the electron affinity (EA) [37,38] first ionization potential (IP) [37,38], chemical hardness (*η*) [39] and softness (*S*) [38], chemical potential (*μ*) [40], electronegativity (*χ*) [41,42], and the electrophilicity index (*ω*) [40], which are linked to their electronic structure. The chemical potential, hardness, and electronegativity are defined as follows

(5) $$\mu ={\left(\frac{\partial E}{\partial N}\right)}_{\nu }$$

(6) $$\eta =\frac{1}{2}{\left(\frac{\partial^{2}E}{\partial N^{2}}\right)}_{\nu }=\frac{1}{2}{\left(\frac{\partial \mu }{\partial N}\right)}_{\nu },\;$$

(7) $$\chi =-\mu =-{\left(\frac{\partial E}{\partial N}\right)}_{\nu },\;$$

where *E* and ν are the electronic energy and the constant external potential of an *N*-electron system. As one can see from Equation (6), chemical hardness is the resistance of the chemical potential to change in the number of electrons, i.e., it describes the resistance of the system to exchanging an electronic charge with the environment [38]. The concept of electronegativity was introduced by Pauling as the power of an atom in a molecule to attract an electron to it. To calculate these parameters, the Koopmans’ theorem for closed-shell molecular systems was used [43]. According to this theorem, the ionization potential and electron affinity can be defined as the negative of the highest occupied molecular orbital (HOMO) $\varepsilon_{H}$ and lowest unoccupied molecular orbital (LUMO) $\varepsilon_{L}$ energy, respectively. Electron affinity refers to the capacity of the molecular systems to accept one electron from a donor, while the ionization potential refers to the ability of a molecular system to lose electrons. Thus, the use of the finite difference approximation and Koopmans’ theorem leads to the following expression for the chemical potential

(8) $$\mu =-\frac{1}{2}\left(\mathrm{IP}+\mathrm{EA}\right)=\frac{1}{2}\left(\varepsilon_{L}+\varepsilon_{H}\right).\;$$

In addition, hardness can be calculated as

(9) $$\eta =\frac{1}{2}\left(\varepsilon_{L}-\varepsilon_{H}\right)=\frac{1}{2}\Delta_{\mathrm{HL}},\;$$

where $\Delta_{\mathrm{HL}}=\varepsilon_{L}-\varepsilon_{H}$ is the HOMO–LUMO gap. Note that the maximum electronic charge that the system can accept is $\Delta N_{\max}=-\mu /\eta$ [40]. Softness is logically the reciprocal of hardness [38]

(10) $$S=\frac{1}{2\eta }.\;$$

The electrophilicity concept was proposed by Parr and co-workers, and the electrophilicity index is defined in [40]

(11) $$\omega =\frac{\mu^{2}}{2\eta }.\;$$

The electrophilicity index measures the stabilization in energy when the system acquires an additional electronic charge from the environment. According to Parr, an analog can be drawn between Equation (11) and the equation for power in classical electricity. Thus, the electrophilicity index can be described as some kind of “electrophilic power”. Since electronegativity is the additive inverse of the chemical potential, the electrophilicity index can be also written as

(12) $$\omega =\frac{\chi^{2}}{2\eta }.\;$$

**Figure 7:**
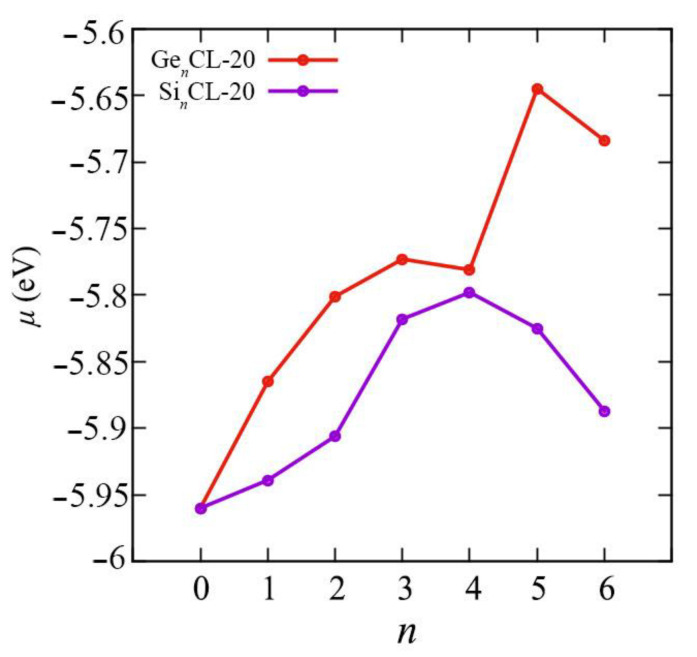
The chemical potential μ versus the number of Si-/Ge-atoms *n* in the Si*_n_*CL-20/Ge*_n_*CL-20 molecules for the most thermodynamically stable configurations obtained at the DFT/B3LYP/6-311(d,p) level of theory.

**Figure 8:**
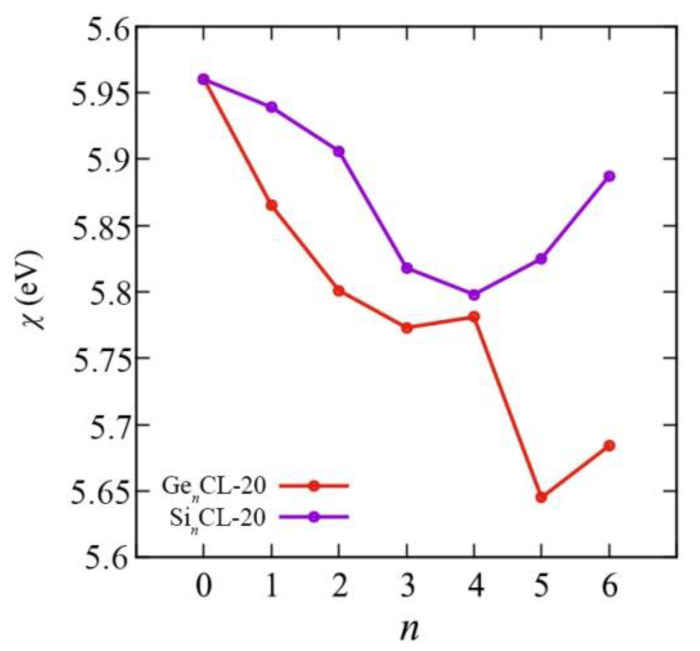
The electronegativity *χ* versus the number of Si-/Ge-atoms *n* in the Si*_n_*CL-20/Ge*_n_*CL-20 molecules for the most thermodynamically stable configurations obtained at the DFT/B3LYP/6-311(d,p) level of theory.

**Figure 9:**
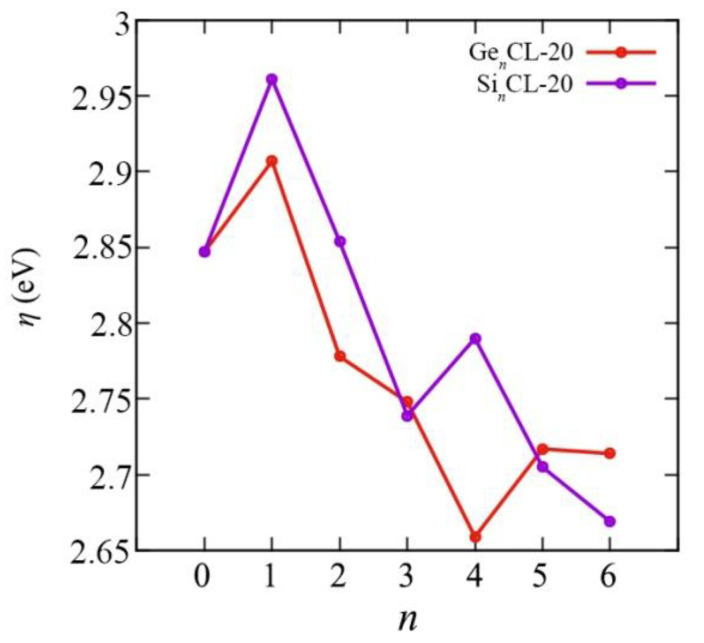
The hardness *η* versus the number of Si-/Ge-atoms *n* in the Si*_n_*CL-20/Ge*_n_*CL-20 molecules for the most thermodynamically stable configurations obtained at the DFT/B3LYP/6-311(d,p) level of theory.

**Figure 10:**
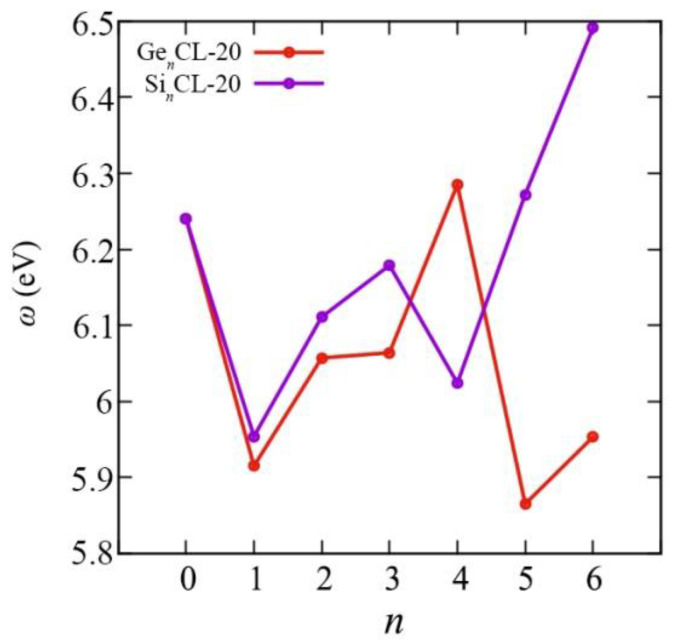
The electrophilicity *ω* versus the number of Si-/Ge-atoms *n* in the Si*_n_*CL-20/Ge*_n_*CL-20 molecules for the most thermodynamically stable configurations obtained at the DFT/B3LYP/6-311(d,p) level of theory.

We obtained all above mentioned quantum–chemical descriptors for the pure CL-20 and the Si*_n_*CL-20/Ge*_n_*CL-20, *n* = 1,…,6. The chemical reactivity parameters are very important indices because they illustrate the reactivity and stability of the compounds. Chemical potential (*μ*), electronegativity (*χ*), hardness (*η*), and the electrophilicity index (*ω*) of the most thermodynamically stable configurations of Si*_n_*CL-20/Ge*_n_*CL-20 are presented in Figure 7, Figure 8, Figure 9 and Figure 10, respectively. More complete data can be found in the Supplementary Materials (Tables S1 and S2).

The electronic chemical potential *μ* characterizes the change in energy when electrons are added to the molecular system, in other words, when the electron density changes. As can be seen from Figure 7, the chemical potential generally rises with the number of substituted atoms *n* in the Ge*_n_*CL-20 system, and for Si*_n_*CL-20 systems, it increases to *n* = 4 and then begins to decrease. Since electronegativity *χ* is defined as the electron chemical potential *μ*, the opposite picture is observed in Figure 8. Thus, with an increase in the number of Ge atoms *n*, the Ge*_n_*CL-20 molecule is less able to attract an electron charge. The smooth monotonic dependence is broken for *n* = 4 and *n* = 6, so that the chemical potential *μ* for Ge_4_CL-20 and Ge_6_CL-20 is −5.781 eV and −5.684 eV, respectively. For silicon-substituted systems, the Si_4_CL-20 molecule is the least capable of attracting an electronic charge; the chemical potential for it is −5.798 eV.

Furthermore, chemical hardness generally decreases with an increasing number of Si/Ge atoms in the system. Therefore, we can say that systems with a large number of germanium atoms are less able to resist a change in electron density for a given change in chemical potential compared to pure CL-20, and a small change in the electron density of the system leads to a smaller change in chemical potential. In this regard, the isolated CL-20 molecule is the most stable in this regard (*η* = 2.907 eV), whereas, with an increase in the number of germanium atoms, Ge*_n_*CL-20 nanostructures become more susceptible to changes in the electronic configuration, except for the case *n* = 1, when the graph experiences a sharp jump. The least stable in this sense is the Ge_4_CL-20 system (*η* = 2.659 eV). For silicon systems, a smooth decay is also broken for *n* = 4.

In addition, it is difficult to establish the exact dependence of electrophilicity on the number of substituted Si-/Ge-atoms; however, analysis of this value shows that Ge_4_CL-20 (among germanium-substituted systems) and Si_6_CL-20 (among silicon-substituted systems) molecules have the highest level of energy stabilization when the system receives an additional charge from the environment.

### 3.5. The CL-20-Based Dimers

**Figure 11:**
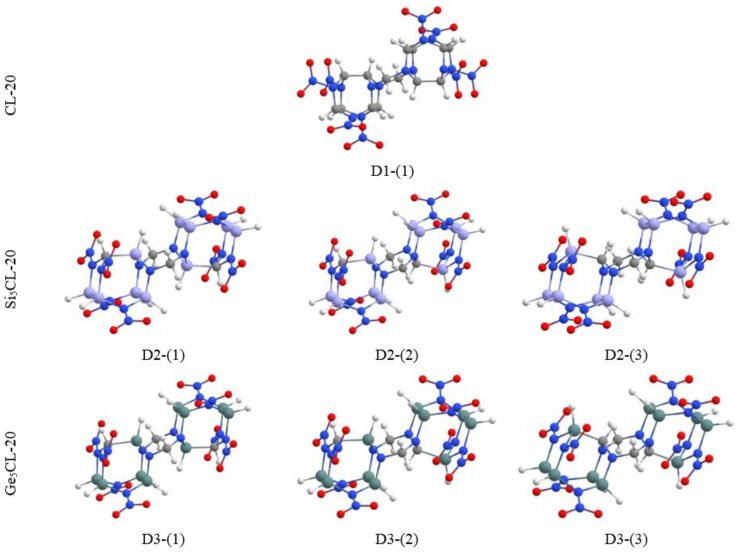
D1-(1)—the dimer of pure molecules CL-20; D2-(1), D2-(2), D2-(3)—the dimers of silicon molecules Si_5_CL-20; D3-(1), D3-(2), D3-(3)—the dimers of germanium molecules Ge_5_CL-20.

In this study, we considered dimers consisting of two CL-20 fragments or their silicon and germanium-substituted analogues, interconnected by CH_2_ functional groups. For the balance between kinetic and thermodynamic stability, the Si_5_CL-20/Ge_5_CL-20 systems were chosen as the derivatives of the CL-20 molecule [21]. Three modifications of such systems were considered, that is, three types of compounds in the fragments of which five silicon or germanium atoms replaced carbon atoms at different positions. The studied systems are shown in Figure 11.

The binding energy *E_b_* of the classical CL-20 dimer, as well as silicon and germanium dimers from Si_5_CL-20/Ge_5_CL-20 fragments, is determined by the equation

(13) $$E_{b}\left[\frac{\mathrm{eV}}{\mathrm{atom}}\right]=\frac{1}{N_{at}}\left\{iE(H)+kE(O)+lE(N)+mE(C)+nE\left(\mathrm{Si}/\mathrm{Ge}\right)-E_{tot}\right\},\;$$

where *N*_at_ = *i* + *k* + *l* + *m* + *n* = 66 is the total number of atoms in the system (*i* = 16; *k* = 16; *l* = 20; *m* = 4; *n* = 10); *E_tot_* is the total energy of the dimer; *E*(H), *E*(O), *E*(N), *E*(O), *E*(Si/Ge) are the energies of the isolated hydrogen, oxygen, nitrogen, carbon, and silicon/germanium atoms, respectively.

The values of the binding energy *E_b_* obtained for the dimers are presented in Table 4. It can be seen that the addition of silicon and germanium atoms to the system leads to a decrease in the binding energy. Thus, the classical CL-20 dimer is more thermodynamically stable compared to its substituted analogues. However, silicon and germanium nanostructures remain stable in this regard.

Dimers based on Ge_5_CL-20 fragments with different types of arrangements of silicon or germanium atoms have close binding energies. Therefore, we can conclude that the arrangement of silicon and germanium atoms in the framework does not strongly affect the thermodynamic stability of the dimer.

The results for HOMO–LUMO gaps, as well as the energy of the highest occupied molecular orbital (HOMO) ε_H_ and the smallest unoccupied molecular orbital (LUMO) ε_L_ systems, are presented in Table 4.

The value of the HOMO–LUMO gap for silicon dimers D2-(1) and D2-(2) is close and less than the corresponding value for the unsubstituted CL-20 dimer. However, for the D2-(3) dimer, on the contrary, the HOMO–LUMO gap is large. For germanium dimers, the HOMO–LUMO gap value is less than the corresponding value for a classical dimer. At the same time, the HOMO–LUMO gaps of germanium dimers differ slightly among themselves. It is assumed that, when building larger systems based on pure CL-20 fragments and their silicon or germanium derivatives, the difference will be more significant. Details on the electronic properties and chemical activity data can be found in the Supplementary Materials (Table S3).

### 3.6. Homodesmotic Reactions and Strain Energies

Compounds in the form of a cage or a ring usually have a high density, and, at the same time, they release a lot of energy when the framework opens. Thus, it is reasonable to evaluate the stability of high-energy compounds from the point of view of the frame deformation energy. The strain of the framework of the compound is associated with the changes in bond distances and bond angles or rotational conformations about the normal hybridization state of the atoms in a molecule [44,45,46]. Cage strain energy makes a great contribution to the power of high-energy compounds. Furthermore, the higher the strain energy characterizing the deformation of the framework, the more unstable the molecule behaves and the more difficult it is to synthesize experimentally [44]. Thus, studies of the strain energy of the cage may be useful for the synthesis of high-energy compounds based on the CL-20 molecule. Strain energy can be defined as the difference between energies for a process that releases strain as determined by the experiment and as obtained from a model that does not involve strain [47].

In this work, we considered several homodesmotic reactions to calculate the strain energies of the high-energy molecule CL-20 and its substituted derivatives. Homodesmotic reactions are a refinement of isodesmic reactions [48] and have been extensively used to calculate strain energy *E_s_* [47,49,50,51,52,53]. In a homodesmotic reaction, the number of bonds of different types and the valence medium around each atom are preserved. The advantage of using a homodesmotic reaction is that the cancellation of errors, associated with the truncation of the basis sets and incomplete electron correlation recovery, occurs to a large extent [49,50]. Thus, the previous studies showed that the theoretically predicted values of *E_s_* in homodesmotic reactions were in good agreement with the experiments [49,52].

**Figure 12:**
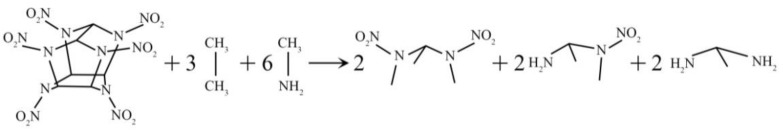
The homodesmotic reaction used to obtain the strain energy of CL-20 framework.

The homodesmotic reaction (Figure 12), previously presented in [44], was used to calculate the strain energies (*E_s_*) of the cells of classical hexanitrohexaazaisowurtzitane CL-20. The same reactions were used to obtain these characteristics for its silicon- and germanium-substituted analogues Si_6_CL-20/Ge_6_CL-20, but carbon atoms were replaced by silicon or germanium atoms in all reactants and reaction products.

Thus, the cage strain energy can be expressed as [38,54]

*E_s_* = *E_tot_(reactants)* − *E_tot_(products)*, (14)

where *E_s_* is the strain energy, *E_tot_(reactants)* is the total energy of the reactants at 0 K, and *E_tot_(products)* is the total energy of the reaction products at 0 K. We ignored the zero-point energies and thermal energy increases ongoing above 0 K. All strain energy values, as well as the total energies of the compounds involved in the homodesmotic reaction necessary for calculation, are presented in Table 5.

Framework strain energy can reflect the thermal stability of high-energy compounds. The higher the strain energy, the more unstable the molecule, and a compound with high cage strain energy is difficult to experimentally synthesize. However, strain is not really a measure of thermodynamic stability, because by itself it does not give a value of the enthalpy or the free energy change for any particular reaction but rather affords a feel for the energy released when the reaction has occurred [54]. An increase of cage strain energy may result in a decrease in stability, but more energy will release when a cage opening occurs [44].

An estimate of the strain energies for a pure CL-20 molecule and its silicon and germanium analogues Si_6_CL-20/Ge_6_CL-20 obtained by homodesmotic reactions shows that the addition of silicon or germanium atoms to the system leads to a decrease in the value of this energy. It indicates that, when the framework of a pure CL-20 molecule opens, most of the energy will be released. Thus, the classical molecule is the most energy-efficient.

## 4. Conclusions

In this work, we studied isolated silicon- and germanium-substituted CL-20 systems and also performed a comparative analysis of their properties compared to a pure unsubstituted CL-20 molecule. Dimers built from the classical CL-20 molecules and their silicon and germanium derivatives Si_5_CL-20/Ge_5_CL-20 were also studied. The energy and electronic properties of Si*_n_*CL-20 and Ge*_n_*CL-20 systems with carbon atoms replaced by silicon/germanium atoms and their dimers were investigated using the B3LYP/6-311G(d,p) level of theory. The addition of silicon/germanium atoms to the system leads to a decrease in the binding energy. However, it has been established that the framework maintains stability with an increase in the number of substituting silicon or germanium atoms. We estimated the value of the strain energy for a CL-20 molecule and its substituted derivatives Si_6_CL-20/Ge_6_CL-20 using homodesmotic reactions and ab initio calculations. It was found that, for a classical pure molecule, more energy will be released when the framework is broken than when the substituted derivatives decay. In this sense, a pure molecule is the most energy-efficient. Therefore, in the future, it is possible to use the studied data of the considered silicon and germanium derivatives of the CL-20 molecule for constructing covalent crystals based on them. The substituted silicon and germanium CL-20 molecules are promising materials since they can be used as a basis for high-energy materials and fuels. The synthesis of such materials is a rather difficult task; therefore, attempts to synthesize new CL-20 molecules should be preceded by comprehensive theoretical studies. Thus, we hope this study will attract more interest from synthesis researchers to explore these compounds.

## Figures and Tables

**Table 1:** The effective diameters *D*_eff_ (Å) of Si*_n_*CL-20/Ge*_n_*CL-20 obtained at the DFT/B3LYP/6-311G(d,p) level of theory.

*n*	0	1	2	3	4	5	6
Si_n_CL-20	3.208	3.363	3.498	3.646	3.803	3.960	4.125
Ge_n_CL-20		3.401	3.556	3.743	3.934	4.142	4.357

**Table 2:** The average bond lengths of the most thermodynamically stable compounds Si*_n_*CL-20 obtained at the DFT/B3LYP/6-311G(d,p) level of theory. A dash “-” means that this bond is not found in the compound.

*n*	C–C, Å	C–N, Å	C–Si, Å	Si–Si, Å	Si–N, Å
0	1.590	1.461	-	-	-
1	1.591	1.461	1.938	-	1.778
2	1.589	1.465	1.941	-	1.778
3	-	1.469	1.944	-	1.779
4	-	1.467	1.940	2.393	1.787
5	-	1.467	1.948	2.372	1.792
6	-	-	-	2.379	1.795

**Table 3:** The average bond lengths of the most thermodynamically stable compounds Ge*_n_*CL-20 obtained at the DFT/B3LYP/6-311G(d,p) level of theory. A dash “-” means that this bond is not found in the compound.

*n*	C–C, Å	C–N, Å	C–Ge, Å	Ge–Ge, Å	Ge–N, Å
0	1.590	1.461	-	-	-
1	1.589	1.460	2.029	-	1.892
2	1.590	1.455	-	2.474	1.902
3	1.593	1.459	2.012	2.473	1.903
4	1.604	1.462	-	2.445	1.914
5	-	1.452	2.017	2.476	1.903
6	-	-	-	2.481	1.903

**Table 4:** The binding energies, HOMO–LUMO gaps, ε_H_ and ε_L_ energies of the dimers based on CL-20, Si_5_CL-20, and Ge_5_CL-20 molecules obtained at the DFT/B3LYP/6-311(d,p) level of theory.

The Dimer		$E_{b},\;\mathrm{eV}/\mathrm{atom}$	*ε*_H_, eV	*ε*_L_, eV	Δ_HL_, eV
D1-(1)		4.156	−7.454	−2.797	4.657
Si	Si-D2-(1)	3.843	−7.489	−3.031	4.458
	Si-D2-(2)	3.843	−7.478	−3.061	4.462
	Si- D2-(3)	3.846	−7.609	−2.293	4.715
Ge	Ge-D3-(1)	3.579	−7.181	−2.771	4.410
	Ge-D3-(2)	3.579	−7.124	−2.780	4.345
	Ge-D3-(3)	3.582	−7.167	−2.793	4.374

**Table 5:** The strain energies *E_s_* of CL-20/Si_6_CL-20/Ge_6_CL-20 molecules obtained from homodesmotic reactions (14) at the DFT/B3LYP/6-311(d,p) level of theory.

Molecule	CL-20	Si_6_CL-20	Ge_6_CL-20
*E_s_*, eV	2.843	1.474	2.039

## Data Availability

The data is available as Supplementary Materials to this article.

## Supplementary Materials

The following supporting information can be downloaded at: https://www.mdpi.com/article/10.3390/ma15196577/s1, Table S1: binding energy and descriptors of reactivity of Si*_n_*CL-20 molecule; Table S2: binding energy and descriptors of reactivity of Ge*_n_*CL-20 molecule; Table S3: binding energies and descriptors of reactivity of covalent dimers based on CL-20, Ge_5_CL-20, and Si_5_CL-20 molecules. Figure S1: The isolated molecule Si_6_CL-20 with numbered silicon atoms. Figure S2: The isolated molecule Ge_6_CL-20 with numbered silicon atoms.
