# Supplementary material for: Energy and Electronic Properties of Nanostructures Based on the CL-20 Framework with the Replacement of the Carbon Atoms by Silicon and Germanium: A Density Functional Theory Study
Source: Materials (Basel). 2022 Sep 22;15(19):6577. doi: 10.3390/ma15196577 (PMC9572965; doi:10.3390/ma15196577)
Supplement: Supplementary file 1 [file materials-15-06577-s001.zip › materials-1920249-supplementary.pdf]

Supplementary Materials
for

Energy and electronic properties of nanostructures based on CL-20 framework with replacement of the carbon atoms by silicon and germanium: A density functional theory study

Margarita A. Gimaldinova[1], Mikhail M. Maslov[1,2,*], Konstantin P. Katin[1,2]

e-mail: Mike.Maslov@gmail.com

[1]Nanoengineering in Electronics, Spintronics and Photonics Institute, National Research Nuclear University “MEPhI”, Kashirskoe Shosse 31, Moscow 115409, Russia

[2]Laboratory of Computational Design of Nanostructures, Nanodevices, and Nanotechnologies, Research Institute for the Development of Scientific and Educational Potential of Youth, Aviatorov str. 14/55, Moscow 119620, Russia

Table S1. Binding energy (E_b), HOMO energy (ε_{H}), LUMO energy (ε_{L}), ionization potential (IP), electron affinity (EA), HOMO-LUMO gap (Δ_{HL}), chemical potential (μ), electronegativity (χ), hardness (η), softness (S), electrophilicity (ω), and maximum electronic charge accepted ($\Delta N_{\max}$) of Si_nCL-20, obtaining at the DFT/B3LYP/6-311G(d,p) level of theory.

Note: n is the number of silicon atoms in the system that replaced the carbon atoms, k shows the numbers of substituted atoms in accordance with the numbering in the figure S1.

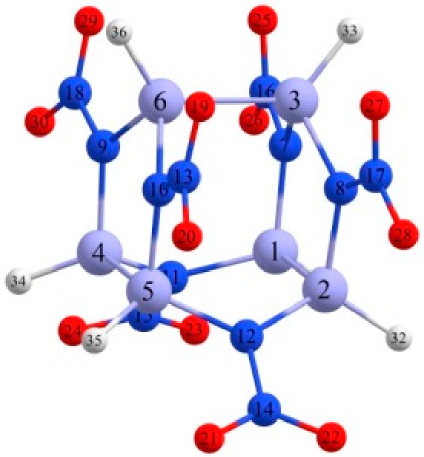

Figure S1. The isolated molecule Si_6CL-20 with numbered silicon atoms.

The structure	*n*	*The number of modification*	*k*	E_b, eV/atom	ε_{H}, eV	ε_{L}, eV	IP, eV	EA, eV	Δ_{HL}, eV	μ, eV	χ, eV	η, eV	S, eV^{-1}	ω, eV	ΔN_{ma}, a.u.
CL-20	**0**	1	–	4.026	-8.807	-3.114	8.807	3.114	5.693	-5.960	5.960	2.847	0.176	6.240	2.094
Si_1CL-20	**1**	1	2	3.970	-8.693	-2.998	8.693	2.998	5.695	-5.845	5.845	2.847	0.176	6.000	2.053
		2	1	3.970	-8.692	-2.998	8.692	2.998	5.694	-5.845	5.845	2.847	0.176	6.000	2.053
		3	3	3.971	-8.900	-2.977	8.900	2.977	5.923	-5.939	5.939	2.961	0.169	5.954	2.005

Si_2CL-20	**2**	1	1,2	3.908	-8.535	-3.009	8.535	3.009	5.525	-5.772	5.772	2.763	0.181	6.029	2.089
		2	1,6	3.913	-8.686	-3.043	8.686	3.043	5.644	-5.865	5.865	2.822	0.177	6.094	2.078
		3	2,6	3.913	-8.687	-3.043	8.687	3.043	5.644	-5.865	5.865	2.822	0.177	6.095	2.078
		4	3,6	3.913	-8.720	-2.955	8.970	2.955	5.765	-5.837	5.837	2.882	0.173	5.910	2.025

Table S1.

The structure	*n*	*The number of modification*	*k*	E_b, eV/atom	ε_H, eV	ε_L, eV	IP, eV	EA, eV	Δ_{HL}, eV	μ, eV	χ, eV	η, eV	*S*, eV^{-1}	ω, eV	ΔN_{ma}, a.u.
		5	1,4	3.914	-8.583	-3.062	8.583	3.062	5.521	-5.822	5.822	2.760	0.181	6.140	2.109
		6	1,5	3.916	-8.658	-2.925	8.658	2.925	5.733	-5.792	5.792	2.866	0.174	5.851	2.020
		7	2,4	3.916	-8.657	-2.924	8.657	2.924	5.733	-5.791	5.791	2.866	0.174	5.849	2.020
		8	1,3	3.921	-8.768	-3.050	8.768	3.050	5.718	-5.909	5.909	2.859	0.175	6.106	2.067
		9	2,3	3.921	-8.759	-3.052	8.759	3.052	5.707	-5.906	5.906	2.854	0.175	6.111	2.070
Si_3CL-20	**3**	1	1,2,6	3.850	-8.509	-2.991	8.509	2.991	5.517	-5.750	5.750	2.759	0.181	5.992	2.084
		2	1,2,5	3.853	-8.642	-3.046	8.642	3.046	5.596	-5.844	5.844	2.798	0.179	6.103	2.089
		3	1,2,4	3.853	-8.638	-3.044	8.638	3.044	5.593	-5.841	5.841	2.797	0.179	6.100	2.089
		4	1,2,3	3.860	-8.657	-3.161	8.657	3.161	5.496	-5.909	5.909	2.748	0.182	6.353	2.150
		5	2,3,6	3.863	-8.635	-3.048	8.635	3.048	5.587	-5.842	5.842	2.793	0.179	6.108	2.091
		6	1,3,6	3.863	-8.636	-3.047	8.636	3.047	5.589	-5.841	5.841	2.794	0.179	6.106	2.090

		7	1,3,5	3.865	-8.625	-2.964	8.625	2.964	5.662	-5.795	5.795	2.831	0.177	5.931	2.047
		8	2,3,4	3.865	-8.627	-2.964	8.627	2.964	5.663	-5.795	5.795	2.831	0.177	5.931	2.047
		9	1,3,4	3.865	-8.560	-3.077	8.560	3.077	5.483	-5.818	5.818	2.742	0.182	6.174	2.122
		10	2,3,5	3.865	-8.556	-3.079	8.556	3.079	5.477	-5.818	5.818	2.739	0.183	6.179	2.124
Si_4CL-20	**4**	1	1,2,4,5	3.794	-8.610	-3.148	8.610	3.148	5.462	-5.879	5.879	2.731	0.183	6.329	2.153
		2	1,2,3,6	3.802	-8.578	-3.127	8.578	3.127	5.452	-5.852	55.852	2.726	0.183	6.282	2.147
		3	1,2,5,6	3.802	-8.497	-3.061	8.497	3.061	5.437	-5.779	5.779	2.718	0.184	6.143	2.126
		4	1,2,4,6	3.803	-8.497	-3.061	8.497	3.061	5.436	-5.779	5.779	2.718	0.184	6.144	2.126
		5	1,2,3,5	3.806	-8.577	-3.116	8.577	3.116	5.462	-5.846	5.846	2.731	0.183	6.257	2.141
		6	1,2,3,4	3.806	-8.575	-3.121	8.575	3.121	5.454	-5.848	5.848	2.727	0.183	6.270	2.144
		7	1,3,4,6	3.814	-8.475	-3.085	8.475	3.085	5.390	-5.780	5.780	2.695	0.186	6.197	2.144
		8	1,3,5,6	3.815	-8.588	-3.007	8.588	3.007	5.581	-5.798	5.798	2.791	0.179	6.023	2.078
		9	2,3,4,6	3.815	-8.588	-3.008	8.588	3.008	5.580	-5.798	5.798	2.790	0.179	6.024	2.078
Si_5CL-20	**5**	1	1,2,3,4,5	3.747	-8.449	-3.205	8.449	3.205	5.243	-5.827	5.827	2.622	0.191	6.476	2.223
		2	1,2,3,4,6	3.755	-8.530	-3.120	8.530	3.120	5.410	-5.825	5.825	2.705	0.185	6.271	2.153
		3	1,2,3,5,6	3.755	-8.530	-3.120	8.530	3.120	5.409	-5.825	5.825	2.705	0.185	6.272	2.154
Si_6CL-20	**6**	1	1,2,3,4,5,6	3.699	-8.556	-3.217	8.556	3.217	5.339	-5.887	5.887	2.669	0.187	6.491	2.205

Table S2. Binding energy (E_b), HOMO energy (ε_H), LUMO energy (ε_L), ionization potential (IP), electron affinity (EA), HOMO-LUMO gap (Δ_{HL}), chemical potential (μ), electronegativity (χ), hardness (η), softness (*S*), electrophilicity (ω), and maximum electronic charge accepted (ΔN_{max}) of Ge_nCL-20, obtaining at the DFT/B3LYP/6-311G(d,p) level of theory.
Note: *n* is the number of germanium atoms in the system that replaced the carbon atoms, *k* shows the numbers of substituted atoms in accordance with the numbering in the figure S1.

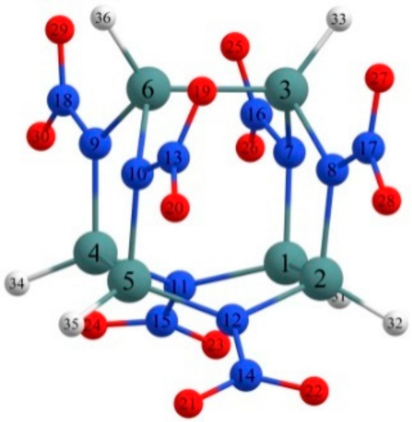

Figure S2. The isolated molecule Ge_6CL-20 with numbered silicon atoms.

The structure	*n*	*The number of modification*	*k*	E_b, eV/atom	ε_H, eV	ε_L, eV	IP, eV	EA, eV	Δ_{HL}, eV	μ, eV	χ, eV	η, eV	*S*, eV^{-1}	ω, eV	ΔN_{max}, a.u.
CL-20	**0**	1	–	4.026	-8.807	-3.114	8.807	3.114	5.693	-5.960	5.960	2.847	0.176	6.240	2.094
Ge_1CL-20	**1**	1	2	3.916	-8.593	-2.943	8.593	2.943	5.650	-5.768	5.768	2.825	0.177	5.889	2.042
		2	1	3.916	-8.591	-2.952	8.591	2.952	5.639	-5.772	5.772	2.819	0.177	5.908	2.047
		3	3	3.920	-8.772	-2.957	8.772	2.957	5.814	-5.865	5.865	2.907	0.172	5.915	2.017
Ge_2CL-20	**2**	1	1,4	3.806	-8.473	-2.976	8.473	2.976	5.497	-5.724	5.724	2.748	0.182	5.961	2.083
		2	2,4	3.808	-8.466	-2.864	8.466	2.864	5.601	-5.665	5.665	2.801	0.179	5.729	2.023
		3	1,5	3.808	-8.467	-2.863	8.467	2.863	5.604	-5.665	5.665	2.802	0.178	5.726	2.022

		4	2,6	3.810	-8.499	-3.115	8.499	3.115	5.384	-5.807	5.807	2.692	0.186	6.263	2.157
		5	1,6	3.810	-8.495	-3.118	8.495	3.118	5.378	-5.807	5.807	2.689	0.186	6.270	2.160

***Table S2*.**

The structure	*n*	*The number of modification*	*k*	E_b, eV/atom	ε_{H}, eV	ε_{L}, eV	IP, eV	EA, eV	Δ_{HL}, eV	μ, eV	χ, eV	η, eV	*S*, eV^{-1}	ω, eV	ΔN_{max}, a.u.
		6	1,3	3.815	-8.627	-2.925	8.627	2.925	5.702	-5.776	5.776	2.851	0.175	5.851	2.026
		7	2,3	3.815	-8.627	-2.925	8.627	2.925	5.703	-5.776	5.776	2.851	0.175	5.850	2.026
		8	1,2	3.818	-8.482	-3.036	8.482	3.036	5.446	-5.759	5.759	2.723	0.184	6.090	2.115
		9	3,6	3.823	-8.579	-3.023	8.579	3.023	5.556	-5.801	5.801	2.778	0.180	6.057	2.088
Ge_3CL-20	**3**	1	1,3,5	3.705	-8.411	-3.018	8.411	3.018	5.393	-5.714	5.714	2.697	0.185	6.055	2.119
		2	2,3,4	3.705	-8.411	-3.019	8.411	3.019	5.391	-5.715	5.715	2.696	0.185	6.058	2.120
		3	2,3,5	3.706	-8.371	-2.955	8.371	2.955	5.417	-5.663	5.663	2.708	0.185	5.920	2.090
		4	1,3,4	3.706	-8.353	-2.947	8.353	2.947	5.406	-5.650	5.650	2.703	0.185	5.906	2.090
		5	1,2,6	3.713	-8.193	-3.138	8.193	3.138	5.055	-5.665	5.665	2.528	0.198	6.349	2.241
		6	1,2,4	3.714	-8.276	-3.001	8.276	3.001	5.274	-5.639	5.639	2.637	0.190	6.028	2.138
		7	1,2,5	3.714	-8.276	-3.001	8.276	3.001	5.275	-5.638	5.638	2.638	0.190	6.027	2.138
		8	1,2,3	3.719	-8.494	-2.990	8.494	2.990	5.504	-5.742	5.742	2.752	0.182	5.990	2.086
		9	1,3,6	3.719	-8.521	-3.024	8.521	3.024	5.498	-5.773	5.773	2.749	0.182	6.061	2.100
		10	2,3,6	3.719	-8.522	-3.025	8.522	3.025	5.496	-5.773	5.773	2.748	0.182	6.064	2.101
Ge_4CL-20	**4**	1	1,2,3,5	3.610	-8.303	-2.930	8.303	2.930	5.373	-5.617	5.617	2.687	0.186	5.871	2.091
		2	1,2,3,4	3.612	-8.299	-2.994	8.299	2.994	5.305	-5.646	5.646	2.652	0.189	6.010	2.129
		3	1,2,4,6	3.612	-8.193	-3.122	8.193	3.122	5.071	-5.657	5.657	2.536	0.197	6.311	2.231
		4	1,2,5,6	3.613	-8.179	-3.074	8.179	3.074	5.104	-5.626	5.626	2.552	0.196	6.202	2.205
		5	1,3,4,6	3.617	-8.354	-2.927	8.354	2.927	5.427	-5.640	5.640	2.714	0.184	5.861	2.078
		6	2,3,4,6	3.618	-8.367	-2.904	8.367	2.904	5.463	-5.636	5.636	2.732	0.183	5.814	2.063
		7	1,3,5,6	3.618	-8.373	-2.901	8.373	2.901	5.472	-5.637	5.637	2.736	0.183	5.807	2.060
		8	1,2,4,5	3.622	-8.217	-3.185	8.217	3.185	5.032	-5.701	5.701	2.516	0.199	6.459	2.266
		9	1,2,3,6	3.623	-8.441	-3.122	8.441	3.122	5.318	-5.781	5.781	2.659	0.188	6.285	2.174
Ge_5CL-20	**5**	1	1,2,3,4,5	3.512	-8.124	-3.027	8.124	3.027	5.097	-5.576	5.576	2.549	0.196	6.099	2.187
		2	1,2,3,5,6	3.518	-8.369	-2.919	8.369	2.919	5.449	-5.644	5.644	2.725	0.184	5.845	2.071

		3	1,2,3,4,6	3.518	-8.362	-2.929	8.362	2.929	5.433	-5.645	5.645	2.717	0.184	5.865	2.078
Ge_6CL-20	**6**	1	1,2,3,4,5,6	3.426	-8.398	-2.971	8.398	2.971	5.427	-5.684	5.684	2.714	0.184	5.953	2.095

Table S3. Binding energy (E_b), HOMO energy (ε_H), LUMO energy (ε_L), ionization potential (IP), electron affinity (EA), HOMO-LUMO gap (Δ_{HL}), chemical potential (μ), electronegativity (χ), hardness (η), softness (*S*), electrophilicity (ω), and maximum electronic charge accepted (ΔN_{max}) of covalent dimers of the CL-20, Ge5CL-20 and Si5CL-20 systems, obtaining at the DFT/B3LYP/6-311G(d,p) level of theory.

The dimer		E_b, eV/atom	ε_H, eV	ε_L, eV	IP, eV	EA, eV	Δ_{HL}, eV	μ, eV	χ, eV	η, eV	*S*, eV^{-1}	ω, eV	ΔN_{max}, a.u.
D1-(1)		4.156	-7.454	-2.797	7.454	2.797	4.657	-5.126	5.126	2.328	0.215	5.641	2.201
Si	D2-(1)	3.843	-7.489	-3.031	7.489	3.031	4.458	-5.260	5.260	2.229	0.224	6.207	2.360
	D2-(2)	3.843	-7.478	-3.061	7.478	3.061	4.462	-5.247	5.247	2.231	0.224	6.170	2.352
	D2-(3)	3.846	-7.609	-2.293	7.609	2.293	4.715	-5.251	5.251	2.358	0.212	5.848	2.227
Ge	D3-(1)	3.579	-7.181	-2.771	7.181	2.771	4.410	-4.976	4.976	2.205	0.227	5.614	2.257
	D3-(2)	3.579	-7.124	-2.780	7.124	2.780	4.345	-4.952	4.952	2.172	0.230	5.645	2.280
	D3-(3)	3.582	-7.167	-2.793	7.167	2.793	4.374	-4.980	4.980	2.187	0.229	5.670	2.277
